# Development and validation of a cancer-associated fibroblast-derived lncRNA signature for predicting clinical outcomes in colorectal cancer

**DOI:** 10.3389/fimmu.2022.934221

**Published:** 2022-07-29

**Authors:** Hongda Pan, Jingxin Pan, Jianghong Wu

**Affiliations:** ^1^ Department of Gastric Surgery, Fudan University Shanghai Cancer Center, Shanghai, China; ^2^ Department of Oncology, Shanghai Medical College, Fudan University, Shanghai, China; ^3^ Department of Hematology, The Second Affiliated Hospital of Fujian Medical University, Quanzhou, China

**Keywords:** cancer-associated fibroblasts, long non-coding RNAs, colorectal cancer, pan-cancer, prognosis, signature

## Abstract

Cancer-associated fibroblasts (CAFs) are actively involved in cancer progression through generating extracellular matrix and orchestrating the crosstalk within the tumor microenvironment (TME). This study aimed to develop and validate a CAF-derived lncRNA (long non-coding RNA) (CAFDL) signature for predicting clinical outcomes in colorectal cancer (CRC). Clinical data and transcriptomic profiles of 2,320 patients with CRC from The Cancer Genome Atlas (TCGA)-COAD and TCGA-READ datasets and 16 Gene Expression Omnibus datasets were included in this study. CAFDLs were identified using weighted gene co-expression network analysis. The CAFDL signature was constructed using the least absolute shrinkage and selection operator analysis in the TCGA-CRC training set. Multiple CRC cohorts and pan-cancer cohorts were used to validated the CAFDL signature. Patients with high CAFDL scores had significantly worse overall survival and disease-free survival than patients with low CAFDL scores in all CRC cohorts. In addition, non-responders to fluorouracil, leucovorin, and oxaliplatin (FOLFOX)/fluorouracil, leucovorin, and irinotecan (FOLFIRI) chemotherapy, chemoradiotherapy, bevacizumab, and immune checkpoint inhibitors had significantly higher CAFDL scores compared with responders. Pan-cancer analysis showed that CAFDL had prognostic predictive power in multiple cancers such as lung adenocarcinoma, breast invasive carcinoma, stomach adenocarcinoma, and thyroid carcinoma. The CAFDL signature was positively correlated with transforming growth factor-beta (TGF-β) signaling, epithelial–mesenchymal transition, and angiogenesis pathways but negatively correlated with the expression of immune checkpoints such as PDCD1, CD274, and CTLA4. The CAFDL signature reflects CAF properties from a lncRNA perspective and effectively predicts clinical outcomes in CRC and across pan-cancer. The CAFDL signature can serve as a useful tool for risk stratification and provide new insights into the underlying mechanisms of CAFs in cancer immunity.

## Introduction

Colorectal cancer (CRC) is the third most common cancer and the second leading cause of cancer-related death worldwide. Standard treatments for CRC include surgery, adjuvant or neoadjuvant chemotherapy and radiotherapy, and targeted therapy (1). In recent years, immune checkpoint inhibitors (ICIs) have revolutionized the treatment of patients with CRC, especially those with microsatellite instability-high (MSI-H)/mismatch-repair-deficient (dMMR) status (2). Cancer-associated fibroblasts (CAFs) are the most abundant of all stromal cells that populate the tumor microenvironment (TME). CAFs modulate the biological properties of cancer cells and other stromal cells through orchestrating the crosstalk within TME and releasing a variety of regulatory factors (3). The extracellular matrix remodeled by CAFs acts as a physical barrier supporting tumor cell invasion and inhibiting infiltration of antitumor leukocytes, leading to cancer progression, immune evasion, and immunotherapy resistance (4). In addition, CAFs may confer substantial therapeutic resistance by impairing drug delivery and immune signaling pathways (5). Previous studies have shown that high CAF infiltration indicates poor survival. CAFs are identified by protein biomarkers such as alpha–smooth muscle actin or fibroblast activation protein (6). Herrera et al. recently reported a CAF-derived gene signature for predicting CRC prognosis involving 596 protein-coding genes (7). Accumulating evidence suggests that long non-coding RNAs (lncRNAs), a subset of non-coding RNAs with >200 nucleotides in length, are closely implicated in the biological behaviors of CAFs (8, 9). However, comprehensive analysis of lncRNAs associated with CAFs is still lacking. Therefore, studies revealing the roles of CAF in cancer immunology from a lncRNA perspective are warranted. CAFs have a higher infiltration level in CRC compared with other cancer types, suggesting that CAFs play a more important role in CRC than in other cancers. CRC has a large number of high-quality sequencing datasets containing lncRNA expression profiles.

**Figure 1:**
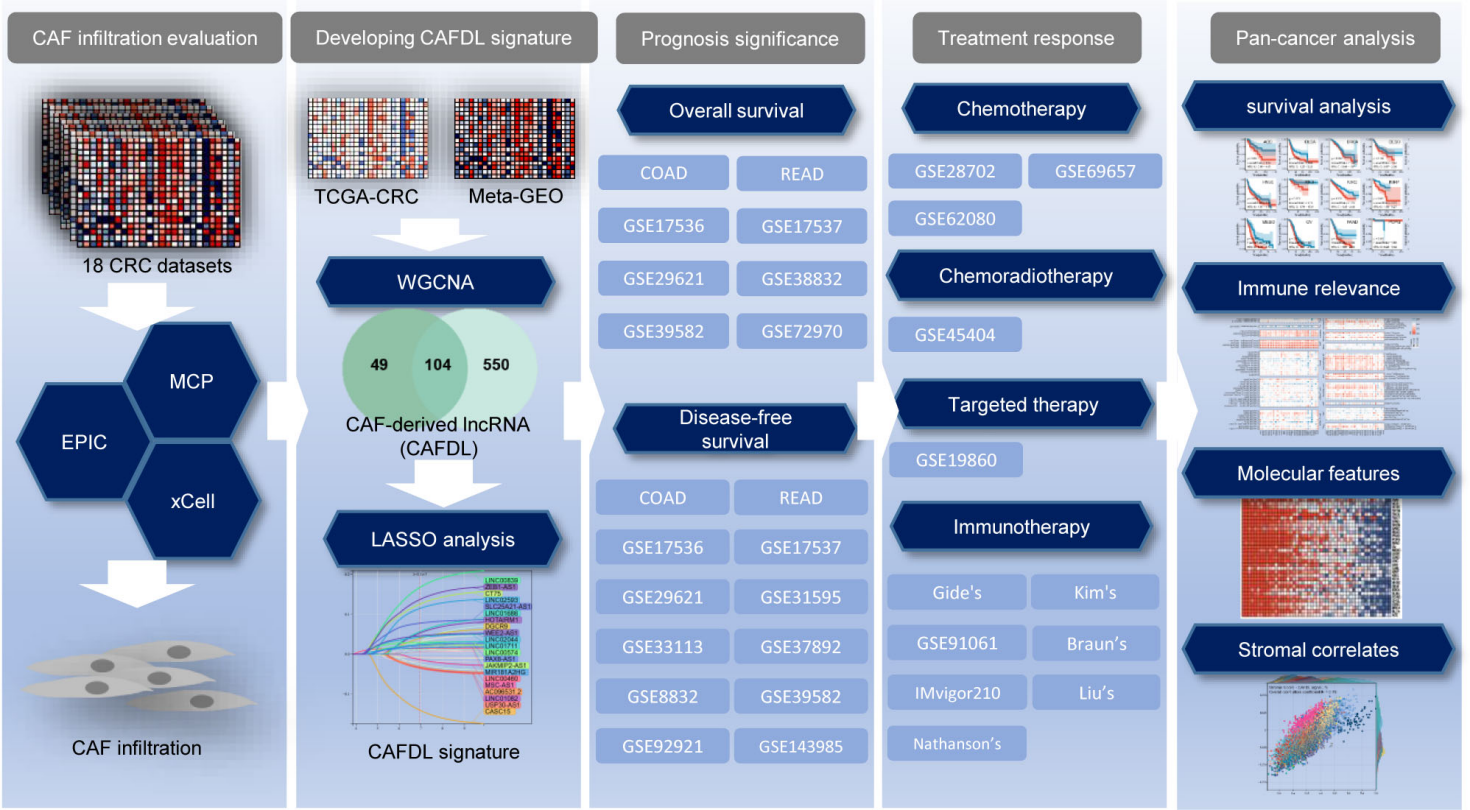
Flow chart of this study.

In this study, we developed and validated a CAF-derived lncRNA (CAFDL) signature based on clinical data and transcriptomic profiles of 2,320 patients with CRC from 18 datasets. The CAFDL signature could serve as a robust predictor of overall survival (OS) and disease-free survival (DFS), as well as response to all mainstay treatments of CRC, including chemotherapy, chemoradiotherapy, targeted therapy, and immunotherapy. Moreover, pan-cancer analysis revealed the predictive power of the CAFDL signature in multiple cancers, and its molecular and immune correlates were explored (**Figure 1**). Our study opens up new avenues for risk stratification and provides new insights into the underlying mechanisms of CAFs in CRC and across pan-cancer.

## Materials and methods

### Data acquisition and processing

Transcriptomic RNA sequencing and corresponding clinical data of 10,148 patients across 33 cancer types including colon adenocarcinoma (COAD) and rectal adenocarcinoma (READ) were downloaded from the TCGA database (https://portal.gdc.cancer.gov). The raw read count was converted to transcripts per kilobase million (TPM) format and log2(x+1)-transformed. Expression profiles and clinical information obtained from the Gene Expression Omnibus (GEO) for 16 CRC datasets (GSE17536, GSE17537, GSE19860, GSE28702, GSE29621, GSE31595, GSE33113, GSE37892, GSE38832, GSE39582, GSE45404, GSE62080, GSE69657, GSE72970, GSE92921, and GSE143985) using the Affymetrix^®^ GPL570 platform. For immunotherapy cohorts, transcriptome and clinical information of IMvigor210 (10) was downloaded from the online database (http://research-pub.gene.com/IMvigor210CoreBiologies). Gene expression profiles and clinical data of Gide’s (11), Nathanson’s (12), Kim’s (13), Braun’s (14), and Liu’s (15) cohorts were obtained from their articles. Expression profiling and clinical data of GSE91061 (16) were downloaded from the GEO database. The “ComBat” tool from the “sva” package of the R software was applied to correct for systematic batch effects among the TCGA and GEO datasets. The “ComBat” tool from the “sva” package of the R software was applied to correct for systematic batch effects between the TCGA-COAD and TCGA-READ datasets and among 16 GEO datasets, respectively. Patients with a follow-up or survival duration of less than 30 days were excluded from survival analysis to rule out the bias due to loss to follow-up or perioperative death.

### Tumor immune microenvironment analysis

CAF infiltrations were evaluated using three algorithms: EPIC (17), xCELL (18), and MCPcounter (19). Tumor purity and the presence of infiltrating stromal/immune cells in tumor tissues were predicted using ESTIMATE algorithm (20). Immune cell infiltrations in 33 cancer types were calculated using seven algorithms: TIMER (21), EPIC, xCELL, CIBERSORT (22), QUANTISEQ (23), MCPcounter, and TIDE (24).

### Weighted gene co-expression network analysis

Weighted gene co-expression network analysis (WGCNA) is a systematic bioinformatics algorithm capable of integrating highly coordinated expressed genes into several gene modules and investigating the relationship of modules to phenotypes of interest. An appropriate soft power threshold (β) was chosen to find the best balance to generate the largest number of modules without loss of gene module membership (MM). WGCNA was conducted using the “WGCNA” package in R.

### Construction of the prognostic signature

The TCGA-CRC cohort was randomly divided into a training set and an internal validation set in a 1:1 ratio. All CAFDLs identified from WCGNA were included in the least absolute shrinkage and selection operator (LASSO) Cox regression model to construct the powerful prognostic signature. LASSO analysis was repeated for 1,000 iterations until the area under the curve (AUC) of time-dependent receiver operating characteristic (ROC) analysis reached a maximum value in both the training and internal test cohorts. A multivariate Cox regression model was finally used to determine the coefficient and construct a prognostic signature based on the candidate lncRNAs generated from the LASSO analyses. A risk score for each patient was calculated as the sum of each gene’s score, which was obtained by multiplying the expression of each gene and its coefficient. The sensitivity and specificity of the prognostic signature were accessed by ROC curves and area under the ROC curves (AUC values).

### Single-sample gene set enrichment analysis

The enrichment scores of cancer hallmark gene sets were calculated by single-sample gene set enrichment analysis (ssGSEA) method with the “ssGSEA” package in R. Cancer hallmark gene sets were downloaded from Molecular Signatures Database.

### Quantitative real-time PCR

TRIzol reagent (Thermo Fisher Scientific, Carlsbad, CA, USA) was used to extract the total RNA from CRC and normal tissues according to the manufacturer’s protocol. Reverse transcription was performed using a Prime Script RT reagent kit (Takara Biotechnology, China). Applied Biosystems 7900 Real-time PCR System (Thermo Fisher Scientific) was used to perform the quantitative real-time PCR (qRT-PCR) assay. glyceraldehyde 3-phosphate dehydrogenase (GAPDH) was used to normalize lncRNA expression.

## Results

### Assessing CAF infiltrations in CRC cohorts

First, we established two integrated cohorts, namely, TCGA-CRC and meta-GEO. The TCGA-CRC cohort of 625 patients consisted of TCGA-COAD (N = 458) and TCGA-READ (N = 167) datasets. On the other hand, the meta-GEO cohort of 1,116 patients was pooled from six GEO datasets with OS data: GSE17536 (N = 177), GSE17537 (N = 55), GSE29621 (N = 65), GSE38832 (N = 122), GSE39582 (N = 573), and GSE72970 (N = 124). CAF infiltrations in each CRC sample were evaluated using three algorithms: EPIC, MCPcounter, and xCELL (**Table S1**).

### WGCNA identified CAFDLs

**Figure 2:**
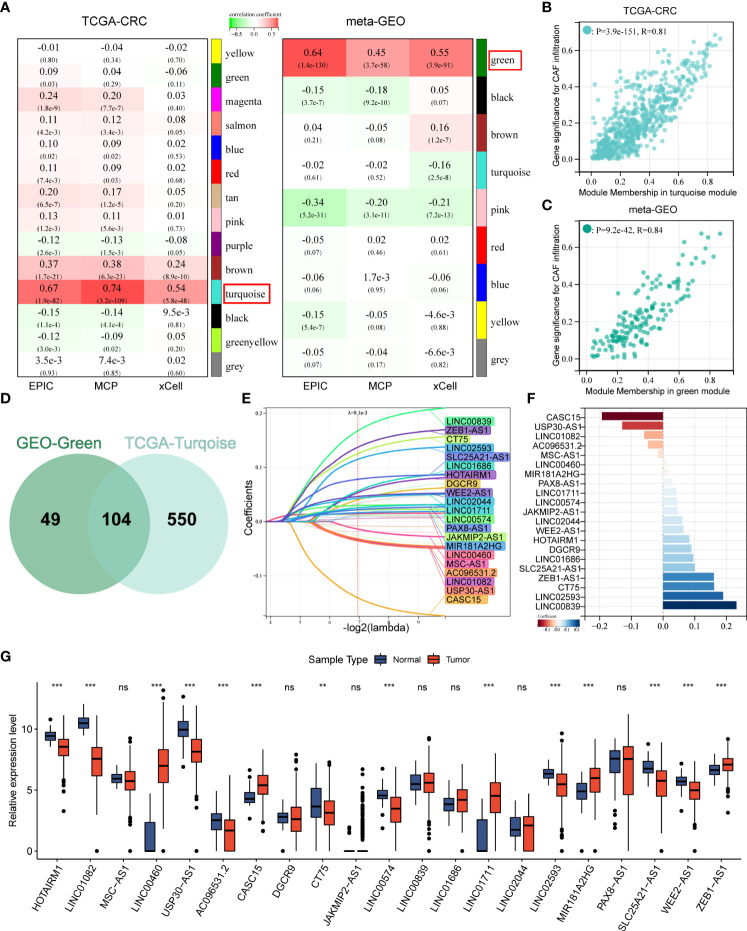
WGCNA identified CAFDL and LASSO analysis. **(A)** WGCNA identified modules associated with CAF infiltration calculated by EPIC, MCPcounter, and xCell in TCGA-CRC and meta-GEO cohorts. **(B)** Correlation between gene significance for CAF infiltration and module membership in turquoise module in TCGA-CRC cohort. **(C)** Correlation between gene significance for CAF infiltration and module membership in green module in meta-GEO cohort. **(D)** A Venn diagram showing the number of lncRNAs in the turquoise module in the TCGA-CRC cohort and the green module in the meta-GEO cohort. **(E)** LASSO analysis identifies 21 CAF-derived lncRNAs. **(F)** Multivariate Cox analysis calculated the coefficient for each lncRNA in the CAFDL signature. **(G)** Expression of 21 CAF-derived lncRNAs in CRC and normal tissues. **P < 0.01, ***P < 0.001, NS non-significant.

After gene symbol annotation, 12,644 lncRNAs in the TCGA-CRC and 2,023 lncRNAs in the meta-GEO cohort were obtained. A total of 1,993 lncRNAs were shared by both cohorts. We performed WGCNA on the lncRNA expression profiles of TCGA-CRC and meta-GEO cohorts, respectively. The optimal soft threshold used to generate modules was 3 for both cohorts. The numbers of modules identified by WGCNA for TCGA-CRC and meta-GEO cohorts were 14 and 9, respectively (**Figure 2A**). We analyzed the relationship between modules and CAF infiltrations assessed by EPIC, MCPcounter, and xCell algorithms. CAF infiltration was significantly associated with turquoise module in TCGA-CRC (R_EPIC_ = 0.67, R_MCP_ = 0.74, and R_xCell_ = 0.54, respectively) (**Figure 2A**). The correlation coefficient between the gene significance (GS) of CAF infiltration and MM in the TCGA-CRC turquoise module reached 0.81 (**Figure 2B**). In meta-GEO, CAF infiltration was significantly associated with green module (R_EPIC_ = 0.64, R_MCP_ = 0.45, and R_xCell_ = 0.55, respectively) (**Figure 2A**). The correlation coefficient between GS of CAF infiltration and MM in the meta-GEO green module reached 0.84 (**Figure 2C**). The turquoise module of TCGA-CRC contains 153 lncRNAs, whereas the green module of meta-GEO contains 654 lncRNAs. We obtained 703 lncRNAs in these two modules, which were defined as CAFDLs (**Figure 2D**).

### Development of the CAFDL signature

The TCGA-CRC cohort was randomly divided into a training set and an internal validation set. LASSO regression analysis was used to select the optimal CAFDLs for building a risk prediction model (**Figure 2E**). A multivariate Cox regression model was finally used to determine the coefficient and construct a prognostic signature based on the candidate lncRNAs generated from the LASSO analyses (**Figure 2F**). The CAFDL signature consists of 21 lncRNAs (HOTAIRM1, LINC01082, MSC-AS1, LINC00460, USP30-AS1, AC096531.2, CASC15, DGCR9, CT75, JAKMIP2-AS1, LINC00574, LINC00839, LINC01686, LINC01711, LINC02044, LINC02593, MIR181A2HG, PAX8-AS1, SLC25A21-AS1, WEE2-AS1, and ZEB1-AS1), and its corresponding risk score (CAFDL Score) is the sum of the products of all lncRNA expression values and coefficients. We examined the expression of these 21 lncRNAs in CRC and normal tissues. Among the 21 lncRNAs, 14 lncRNAs (HOTAIRM1, LINC01082, LINC00460, USP30-AS1, AC096531.2, CASC15, CT75, LINC00574, LINC01711, LINC02593, MIR181A2HG, SLC25A21-AS1, WEE2-AS1, and ZEB1-AS1) were significantly differentially expressed between CRC and adjacent normal tissues. LINC00460, CASC15, LINC01711, MIR181A2HG, and ZEB1.AS1 were significantly upregulated in CRC tissues, whereas the remaining lncRNAs were significantly downregulated in CRC compared with normal tissues (**Figure 2G**). Next, we analyzed the OS and DFS of patients with CRC with high or low expression of the 21 lncRNAs, as suggested by the reviewers. CT75, DGCR9, HOTAIRM1, LINC00460, LINC01082, LINC01711, LINC02044, USP30-AS1, and ZEB1.AS1 were significantly associated with OS (**Figure S1A**), and AC096531.2, CT75, DGCR9, HOTAIRM1, LINC00839, LINC01082, LINC02044, LINC02593, MIR181A2HG, SLC25A21-AS1, WEE2-AS1, and ZEB1.AS1 were significantly associated with DFS (**Figure S1B**).

**Figure 3:**
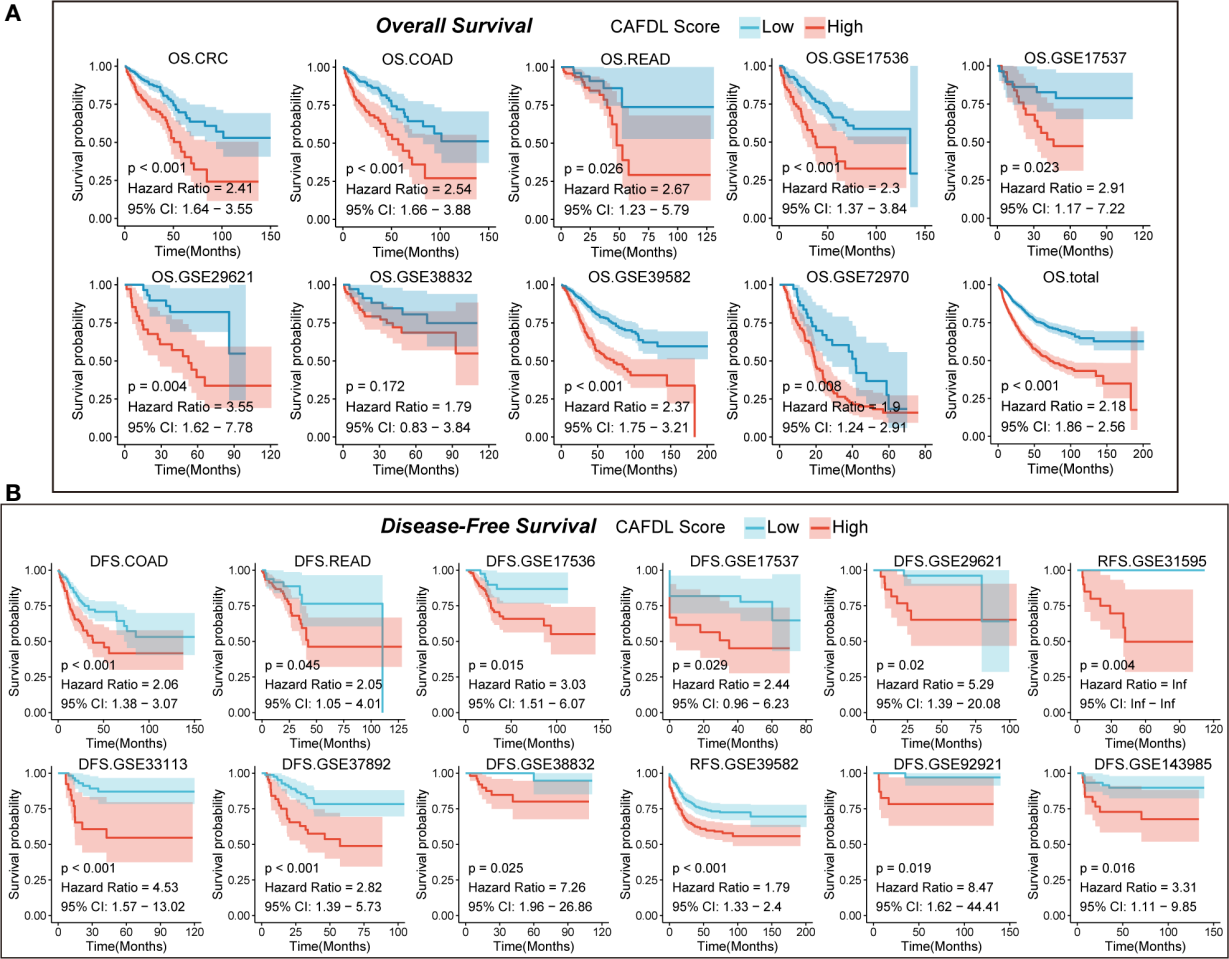
CAFDL signature can effectively predict the prognosis of patients with CRC. **(A)** Patients with high CAFDL scores have significantly worse overall survival than those with low CAFDL scores in TCGA-CRC, TCGA-COAD, TCGA-READ, GSE17536, GSE17537, GSE29621, GSE38832, GSE39582, GSE72970, and total CRC cohorts. **(B)** Patients with high CAFDL scores have significantly worse disease-free survival than those with low CAFDL scores in TCGA-COAD, TCGA-READ, GSE17536, GSE17537, GSE29621, GSE31595, GSE33113, GSE37892, GSE38832, GSE39582, GSE92921, and GSE143985 cohorts.

Each cohort was divided into high and low CAFDL groups according to the optimal cutoff value calculated by the “survminer” package in R. Kaplan–Meier survival analysis showed that patients with high CAFDL scores in the TCGA-CRC cohort had significantly worse OS than patients with low CAFDL scores [P < 0.001, hazard ratio (HR) = 2.41, 95% confidence interval (CI) 1.64–3.55] (**Figure 3A**). We collected 20 pairs of CRC and adjacent normal tissue samples for qRT-PCR analysis. The expression of 11 of 21 lncRNAs (HOTAIRM1, LINC01082, LINC00460, USP30-AS1, CASC15, JAKMIP2-AS1, LINC00574, LINC01711, LINC02593, SLC25A21-AS1, and ZEB1-AS1) was significantly different between CRC and adjacent normal tissues. Among them, LINC00460, CASC15, JAKMIP2-AS1, LINC01711, and ZEB1-AS1 were significantly upregulated in CRC tissues, whereas HOTAIRM1, LINC01082, USP30-AS1, LINC00574, LINC02593, and SLC25A21-AS1 were significantly downregulated in CRC tissues (**Figure S2A**).

### Validation of the predictive value of CAFDL signature for OS in CRC cohorts

We apply the CAFDL signature to eight CRC cohorts to validate its predictive value for OS. In the TCGA-COAD (HR = 2.54, P < 0.001), TCGA-READ (HR = 2.67, P = 0.026), GSE17536 (HR = 2.30, P < 0.001), GSE17537 (HR = 2.91, P = 0.023), GSE29621 (HR = 3.55, P = 0.004), GSE39582 (HR = 2.37, P < 0.001), GSE72970 (HR = 1.90, P = 0.008), and total CRC cohorts (HR = 2.18, P < 0.001), patients with high CAFDL scores had significant worse OS compared with those with low CAFDL scores (**Figure 3A**, **Figure S3**), except for GSE38832 (P = 0.172, HR = 1.79), whose OS difference did not reach statistical significance.

### Validation of the predictive value of CAFDL signature for DFS in CRC cohorts

Next, we validate predictive value of CAFDL signature for DFS in 12 cohort with DFS data. In the TCGA-COAD (HR = 2.06 P < 0.001), TCGA-READ (HR = 2.05, P = 0.045), GSE17536 (HR = 3.03, P = 0.015), GSE17537 (HR = 2.44, P < 0.029), GSE29621 (HR = 5.29, P = 0.02), GSE31959 (HR = infinity, P = 0.004), GSE33113 (HR = 4.53, P < 0.001), GSE37982 (HR = 2.82, P < 0.001), GSE38832 (HR = 7.26, P = 0.025), GSE39582 (HR = 1.79 P < 0.001), GSE92921 (HR = 8.47 P < 0.019), and GSE143982 (HR = 3.31, P = 0.016) cohorts, all patients with high CAFDL scores had significantly worse DFS compared with those with low CAFDL scores (**Figure 3B**). We performed ROC analysis of the CAFDL signature in each of the TCGA and GEO datasets for the predictive ability of DFS and OS at 1, 3, and 5 years and calculated its AUC values (**Figure S2B**).

### CAFDL signature is an independent prognostic factor for OS and DFS

Univariate (**Figures S2C, E**) and multivariate Cox analyses (**Figures S2D, F**) were performed for multiple clinicopathological factors (age, gender, histological differentiation, and American Joint Committee on Cancer (AJCC) TNM stage) together with the CAFDL signature in the TCGA-CRC cohort. The results showed that CAFDL signature, age, and TNM stage were independent prognostic factors for OS, whereas CAFDL signature and TNM stage were independent prognostic factors for DFS.

### CAFDL signature predicts response to chemotherapy, radiotherapy, and targeted therapy

**Figure 4:**
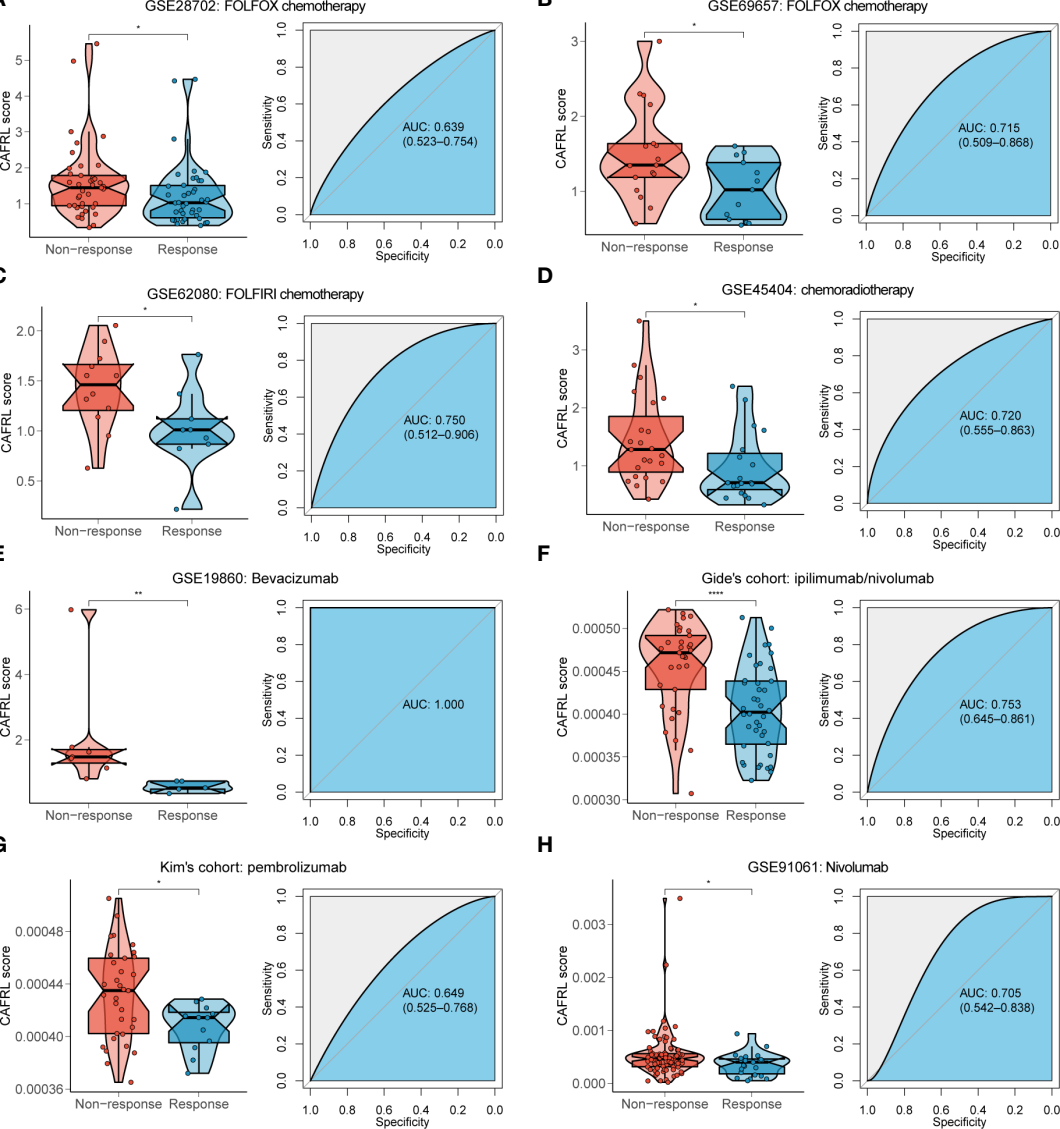
CAFDL signature can effectively predict the response to mainstay treatments of CRC. **(A–E)** Non-responders to FOLFOX **(A, B)** and FOLFIRI **(C)** chemotherapy, chemoradiotherapy **(D)**, and bevacizumab targeted therapy **(E)** had significantly higher CAFDL scores compared with responders (left panels). ROC curves demonstrate the predictive power of the CAFDL signature for response to these treatments (right panels). **(F–H)** Non-responders to ipilimumab/nivolumab **(F)**, pembrolizumab **(G)**, and nivolumab **(H)** had significantly higher CAFDL scores compared with responders (left panels). ROC curves demonstrate the predictive power of the CAFDL signature for response to these treatments (right panels). *P < 0.05, **P < 0.01, and ****P < 0.0001.

Chemotherapy, radiotherapy, and targeted therapy are the mainstay treatments for CRC. Non-responders to FOLFOX (GSE28702 and GSE69657; **Figures 4A, B**) and FOLFIRI (GSE62080; **Figure 4C**) chemotherapy had significantly higher CAFDL scores compared with responders. The AUC values of CAFDL signature for predicting response to chemotherapy in GSE28702 (**Figure 4A**), GSE69657 (**Figure 4B**), and GSE62080 (**Figure 4C**) were 0.639, 0.715, and 0.750, respectively. In addition, CAFDL signature can also effectively predict the response to chemoradiotherapy in patients with rectal cancer (GSE45404, AUC = 0.72); non-responders had significantly higher CAFDL score than responders (**Figure 4D**). Notably, CAFDL signature had excellent predictive power for response to bevacizumab (GSE19860, AUC = 1); all responders belonged to the low CAFDL score group (**Figure 4E**).

### CAFDL signature predicts immunotherapy outcomes

**Figure 5:**
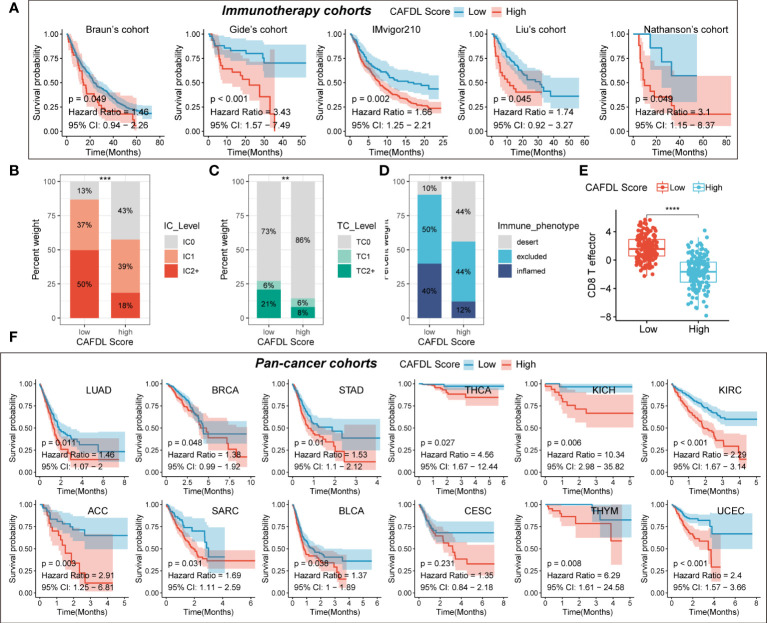
CAFDL signature predicts clinical outcomes in immunotherapy cohorts and pan-cancer cohorts. **(A)** Patients with high CAFDL scores have significantly worse overall survival than those with low CAFDL scores in Braun’s, Gide’s, IMvigor210, Liu’s, and Nathanson’s cohorts. **(B, C)** In the IMvigor210 cohort, patients in the low CAFDL score group had significantly higher PD-L1 protein expression levels in immune cells **(B)** and tumor cells **(C)**. **(D)**The high CAFDL score group had higher proportion of immune desert phenotype and lower proportion of immune-inflamed phenotype. **(E)** The high CAFDL score group had significantly lower CD8^+^ T effector infiltration. **(F)** In addition to COAD and READ, patients with high CAFDL scores have significantly worse overall survival than those with low CAFDL scores in 12 TCGA datasets: LUAD, BRCA, STAD, THCA, KICH, KIRC, ACC, SARC, BLCA, CESC, THYM, and UCEC. **P < 0.01, ***P < 0.001, and ****P < 0.0001.

We applied the CAFDL signature to multiple immunotherapy cohorts and found that non-responders to ICIs had significantly higher CAFDL scores compared with responders in Gide’s cohort (melanoma treated with anti–programmed cell death 1 (PD-1)/cytotoxic T-lymphocyte-associated protein 4 (CTLA-4) antibody; **Figure 4F**), Kim’s cohort (gastric cancer treated with anti–PD-1 antibody; **Figure 4G**), and GSE91061 (melanoma treated with anti–PD-1 antibody; **Figure 4H**). The AUC values of CAFDL signature for predicting response to immunotherapy in Gide’s cohort (**Figure 4F**), Kim’s cohort (**Figure 4G**), and GSE91061 (**Figure 4H**) were 0.753, 0.649, and 0.705, respectively. Moreover, patients with high CAFDL scores had a significantly worse prognosis than those with low CAFDL scores in Braun’s cohort (clear cell renal cell carcinoma treated with anti–PD-1 antibody), Gide’s cohort, IMvigor210 (bladder urothelial carcinoma treated with anti–programmed death ligand 1 (PD-L1) antibody), Liu’s cohort (melanoma treated with anti–PD-1 antibody), and Nathanson’s cohort (melanoma treated with anti–CTLA-4 antibody) (all P < 0.05; **Figure 5A**). In the IMvigor210 cohort, patients in the low CAFDL score group had significantly higher PD-L1 protein expression levels in immune cells (**Figure 5B**) and tumor cells (**Figure 5C**). The high CAFDL score group had higher proportion of immune desert phenotype, lower proportion of immune-inflamed phenotype (**Figure 5D**), and lower CD8^+^ T effector infiltration (**Figure 5E**).

### CAFDL signature predicts prognosis across multiple cancers

In addition to COAD and READ, we also attempted to explore the predictive power of the CAFDL signature for clinical outcomes in other cancers. The CAFDL signature is effective in prognostic stratification in the most common cancers, including lung adenocarcinoma (LUAD), breast invasive carcinoma (BRCA), stomach adenocarcinoma (STAD), thyroid carcinoma (THCA), bladder urothelial carcinoma (BLCA), kidney renal clear cell carcinoma (KIRC), adrenocortical carcinoma (ACC), cervical squamous cell carcinoma and endocervical adenocarcinoma (CESC), kidney chromophobe (KICH), sarcoma (SARC), thymoma (THYM), and uterine corpus endometrial carcinoma (UCEC) (all P < 0.05; **Figure 5F**), implying that CAFDL has broad applicability across pan-cancer.

### Immune correlates of CAFDL signature across pan-cancer

**Figure 6:**
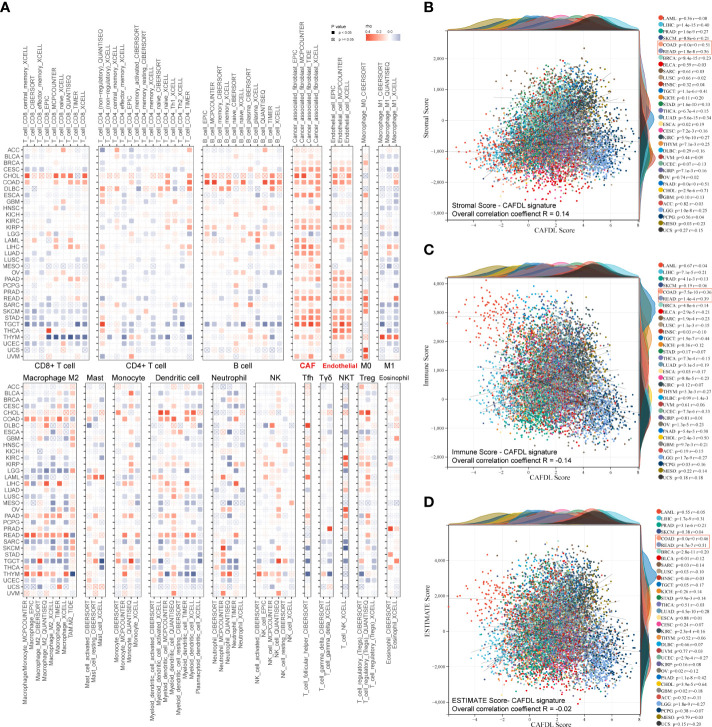
Pan-cancer immune correlates of CAFDL signature. **(A)** Correlation of CAFDL signature with immune cell infiltration evaluated using seven algorithms: TIMER, EPIC, xCELL, CIBERSORT, QUANTISEQ, MCPcounter, and TIDE across pan-cancer. **(B–D)** Correlation of CAFDL signature with stromal score **(B)**, immune score **(C)**, and ESTIMATE score **(D)** across pan-cancer.

To fully demonstrate the pan-cancer TME landscape, immune cell infiltrations across pan-cancer were evaluated using seven algorithms: TIMER, EPIC, xCell, CIBERSORT, QUANTISEQ, MCPcounter, and TIDE (**Figure 6A**). As expected, the CAFDL signature was closely associated with the CAF infiltration (**Figure 6A**). Epithelial cells, another important member of the stromal component, also had a strong correlation with the CAFDL signature. In addition, the CAFDL signature was also significantly associated with macrophage M2 in COAD and READ. CAFDL signature showed no or negative correlation with major immune cells such as CD8^+^/CD4^+^ T cells, B cells, and M1 macrophages.

Next, we used the ESTIMATE algorithm to evaluate the pan-cancer stromal score and immune score. The CAFDL signature showed a positive correlation with the stromal score, with an overall correlation of 0.14 for the entire pan-cancer cohort and a median correlation of 0.16 across 33 cancers, ranging from −0.25 to 0.71 (**Figure 6B**). However, CAFDL exhibited negative correlations with the immune score (R = −0.14; **Figure 6C**) and the ESTIMATE score (the integration of the stromal score and the immune score, R = −0.02; **Figure 6D**), respectively. Notably, CAFDL signature showed moderate correlation with stromal score in COAD (R = 0.51) and READ (R = 0.56) and weak correlation with immune score in COAD (R = 0.36) and READ (R = 0.39), respectively. These results indicated that CAFDL could specifically reflect the properties of stromal components in TME but had a weak correlation with immune cell infiltration.

### Molecular features of CAFDL signature

**Figure 7:**
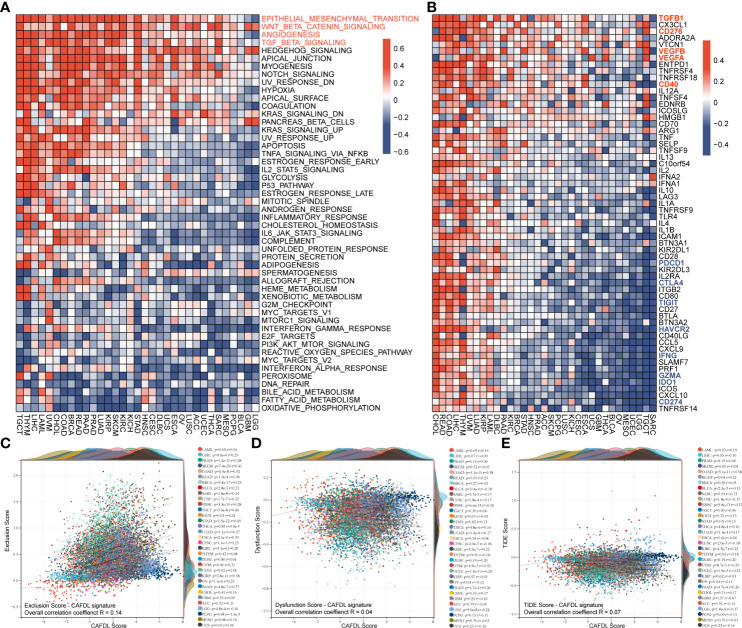
Molecular features of CAFDL signature. **(A)** Correlation of CAFDL signature with cancer hallmark gene sets across pan-cancer. **(B)** Correlation of CAFDL signature with common immune regulators across pan-cancer. **(C–E)** Correlation of CAFDL signature with immune exclusion score **(C)**, immune dysfunction score **(D)**, and TIDE score **(E)** across pan-cancer.

We calculated the enrichment scores for cancer hallmark gene sets across 33 cancer types using the ssGSEA method. The CAFDL signature was significantly positively correlated with epithelial–mesenchymal transition (EMT), WNT/β-Catenin signaling, angiogenesis, and TGF-β signaling pathways across pan-cancer, which are important mechanisms that occur in the tumor stroma to promote tumor development and metastasis (**Figure 7A**). Moreover, we analyzed the correlation of CAFDL signature with expression of immune regulators. TGF-β is well known to be one of the most important regulators of CAF activation (25). The CAFDL signature was significantly positively associated with TGFB1, CD276, CD40, VEGFA, VEGFB, etc., but showed significantly negative correlation with immune checkpoints (such as CD274, PDCD1, CTLA4, TIGHT, and HAVCR2) and anti-cancer immune regulators (IFNG, IDO1, and GZMA) (**Figure 7B**).

### CAFDL signature is associated with immune exclusion

The TIDE online tool was used to assess the potential of immune escape across pan-cancer. The TIDE score consists of two components: immune dysfunction and immune exclusion. CAFDL signature was positively correlated with exclusion score, with an overall correlation of 0.14 for the entire pan-cancer cohort and a median correlation of 0.24 across 33 cancers, ranging from −0.16 to 0.49 (**Figure 7C**). However, CAFDL signature had little correlation with dysfunction score (R = 0.04; **Figure 7D**) and TIDE score (R = 0.07; **Figure 7E**), suggesting that CAF prevents immune cells from killing tumor cells more by generating extracellular matrix (immune exclusion) than by directly causing immune dysfunction.

### CAFDL signature is independent of tumor mutation burden and microsatellite instability

Microsatellite instability (MSI) and tumor mutation burden (TMB) are well-established predictors of response to immunotherapy, but they are both intrinsic features of cancer cells and are theoretically unrelated to CAFs. In the GSE39582, GSE92921, and GSE143985 cohorts, there were no significant differences in CAFDL scores between mutant and wild-type tumors of v-raf murine sarcoma viral oncogene homolog B1 (BRAF) (**Figures S4A–C**), kirsten rat sarcoma viral oncogene (KRAS) (**Figures S4D–F**), and tumor protein P53 (TP53) (**Figures S4G–I**). Moreover, we found little correlation between CAFDL signature and TMB across 33 cancers (**Figure S5A**), including COAD (R = 0.13) and READ (R = 0.02). Likewise, CAFDL scores of MSI-H/dMMR tumors were not significantly different from those of MSS/pMMR tumors in TCGA-COAD, TCGA-READ, GSE39582, GSE92921, and GSE143985 cohorts (**Figures S5B–F**).

## Discussion

CAFs are major components of the TME and interact with cancer cells by secreting extracellular matrix proteins as well as cytokines and growth factors. CAFs block immune cell infiltration and drug delivery, leading to immune escape and resistance to various treatments including chemotherapy, radiotherapy, targeted therapy, and immunotherapy. In recent years, several studies have shown that CAF is closely related to the poor prognosis of patients with cancer (26–28), and the underlying mechanisms have begun to be revealed. Chen et al. reported that CAFs impact the survival outcomes and treatment response in CRC by regulating immune system (27). Li et al. discovered a subgroup of CAFs correlated with poor survival outcomes in patients with gastric cancer using single-cell RNA sequencing (29). Sun et al. demonstrated that prognostic signature based on CAF-secreted cytokines were associated with genetic alterations and clinical outcomes (30). Zheng et al. revealed that CAFs play an important role in TME, and their secreted extracellular protein can serve as a prognostic marker for triple-negative breast cancer (31). However, these studies on CAFs are based on protein-encoding genes, and studies on lncRNAs are still lacking. Herrera et al. (7) reported a CAF-derived gene signature for predicting CRC prognosis involving 596 protein-coding genes rather than lncRNAs, which is different from our study. Zhang et al. (8) found that DNM3OS, a CAF-promoted lncRNA, confers radio-resistance by regulating DNA damage response in esophageal squamous cell carcinoma. This study focused on the biological function of a specific CAF-related lncRNA, whereas our study was a comprehensive analysis of CAF-related lncRNAs. Liu et al. (9) developed an immune-derived lncRNA signature for improving outcomes in CRC using machine learning methods. This study involved immune-derived lncRNAs rather than specifically focusing on CAFDLs. LncRNA signatures have been widely reported in CRC, and these signatures are closely related to specific biological behaviors, including tumor immunity (9), epigenetic modification (32, 33), and cell death (34). To the best of our knowledge, this is the first comprehensive study on CAFDLs in CRC, to establish a CAFDL signature in CRC, which is innovative.

WGCNA has been successfully applied to identify gene modules with various biological functions or cellular characteristics (35, 36). In our study, we used WGCNA to establish a co-expression network of lncRNAs and obtained multiple modules through co-expression relationships. We analyzed the correlation between the expression level of each module and CAF score in CRC tissues, identified CAF-related lncRNA modules, and finally identified CAFDLs.

Many studies have established lncRNA-based prognostic prediction models (37–40). Liu et al. developed a novel immune-related lncRNA signature in endometrial carcinoma (37), patients were randomly divided into training cohort and test cohort, univariate Cox analysis was used to screen lncRNAs associated with prognosis, LASSO regression was used to screen lncRNAs most associated with DFS, and finally multivariate Cox was used to establish a scoring system. In another study developing an EMT-related lncRNA signature (38), patients were also randomly divided into training group and test group, risk prediction model was built, and the weight of each lncRNA was calculated using LASSO regression. Yuan et al. identified m5C-related lncRNAs in pancreatic ductal adenocarcinoma (39), a preliminary screening was performed by univariate Cox, a prediction model was established by LASSO regression, and a risk score was calculated. A recent study constructed a mutation-derived genome instability-related lncRNAs signature in endometrial cancer (40), patients were randomized 1:1 into training or test sets, and risk prediction models were built using univariate and multivariate Cox regression. In our study, we used TCGA-CRC to build a risk prediction model and used the meta-GEO cohort as external validation. The TCGA-CRC cohort is randomly split into a training set and an internal validation set in a 1:1 ratio. The LASSO analysis was repeated for 1,000 iterations until the AUC reached a maximum value in both the training set and the internal test set. Multivariate Cox regression models were finally used to determine coefficients and construct prognostic signatures based on candidate lncRNAs generated by LASSO analysis. In contrast to the previously mentioned literatures, we did not perform a univariate analysis of the initial screening. This is because lncRNAs that constitute prognostic risk models may not reach statistical significance when prognostic analysis is performed on individual genes. Potential prognostic information may be lost if certain important lncRNAs are deleted. Then, because the results of LASSO regression analysis may vary each time, we used multivariate Cox analysis to finally determine the weight coefficient of each lncRNA after LASSO regression established the prognostic model, instead of directly using LASSO regression to calculate the coefficient, which was similar to the analysis method of Liu’s study (37).

Our study included 18 datasets of 2,320 patients with CRC, including COAD and READ datasets from the TCGA database, and 16 CRC datasets from the GEO database. We established the CAFDL signature in TCGA-CRC training set and verified its predictive value in all CRC datasets. The CAFDL signature can effectively predict the prognosis of patients with CRC, including OS and DFS. In addition, CAFDL has also demonstrated robust predictive power for response to chemotherapy, radiotherapy, and targeted therapy, which are the mainstays of treatment for CRC. Seven additional immunotherapy datasets were incorporated into our study, and we found that CAFDL can be used as a predictor of response to ICIs. Through comprehensive analysis based on large-scale clinical samples and transcriptomic data, we demonstrate that CAFDL can serve as a robust tool for predicting survival outcomes and treatment response in patients with CRC.

Furthermore, pan-cancer analysis showed that, in addition to COAD and READ, CAFDL had prognostic predictive power in multiple cancers (such LUAD, BRCA, STAD, and THCA). The expression level of CAFDL in pan-cancer is not clear, and the CAFDL signature may not be applicable in all tumors. The purpose of pan-cancer analysis in our study is to try to expand the applicability of CAFDL signature to other cancers. This provides evidence for researchers to conduct further studies in other cancer types in the future.

We further explored the molecular and immune mechanisms and found that CAFDL signature was positively correlated with TGF-β signaling, EMT, and angiogenesis pathways but negatively correlated with the expression of immune checkpoints such as PDCD1, CD274, and CTLA4. Moreover, the CAFDL signature was independent of MSI and TMB, both of which are intrinsic features of cancer cells rather than stromal cells.

## Conclusion

In summary, we developed the robust CAFDL signature that can effectively predict the survival outcomes and response to multiple treatments in patients with CRC. Our study provides a roadmap for patient stratification and may help improve strategies for personalized follow-up and individualized decision making for CRC.

## Data availability statement

The datasets presented in this study can be found in online repositories. The names of the repository/repositories and accession number(s) can be found in the article/**Supplementary Material**.

## Author contributions

Conception and design: HP and J.W. Acquisition of data: PL. Writing, review, and revision of the manuscript: HP, JP, and PL. Analysis and interpretation of data: HP and JP. Development of methodology: HP and JP. All authors contributed to the article and approved the submitted version.

## Funding

This study was funded by the National Natural Science Foundation of China (81902424).

## Conflict of interest

The authors declare that the research was conducted in the absence of any commercial or financial relationships that could be construed as a potential conflict of interest.

## Publisher’s note

All claims expressed in this article are solely those of the authors and do not necessarily represent those of their affiliated organizations, or those of the publisher, the editors and the reviewers. Any product that may be evaluated in this article, or claim that may be made by its manufacturer, is not guaranteed or endorsed by the publisher.

## Supplementary material

Supplementary Figure 1 Survival analysis for the 21 lncRNAs constituting the CAFDL signature. **(A)** Overall survival. **(B)** Disease-free survival.

Supplementary Figure 2 Validation of the CAFDL signature. **(A)** qRT-PCR detected the expression levels of 21 lncRNAs constituting the CAFDL signature in paired CRC and adjacent normal tissues. **(B)** ROC analysis of the predictive ability of CAFDL signature on DFS and OS at 1, 3, and 5 years in CRC datasets. **(C, D)** Univariate and multivariate Cox analysis identify independent predictive factors for OS in TCGA-CRC cohort. **(E, F)** Univariate and multivariate Cox analysis identify independent predictive factors for DFS in TCGA-CRC cohort.

Supplementary Figure 3 Landmark analysis of GSE17537.

Supplementary Figure 4 CAFDL score in patients with wild-type or mutant BRAF, Kras and TP53. **(A-C)** CAFDL score in patients with wild-type or mutant BRAF in GSE39582, GSE92921 and GSE143985 cohorts. **(D-F)** CAFDL score in patients with wild-type or mutant Kras in GSE39582, GSE92921 and GSE143985 cohorts. **(G-I)** CAFDL score in patients with wild-type or mutant TP53 in GSE39582, GSE92921 and GSE143985 cohorts. ns, non-significant.

Supplementary Figure 5 Correlation between CAFDL signature and TMB and MSI/MMR status. **(A)** Correlation of CAFDL signature with TMB across pan-cancer. **(B, C)** CAFDL scores of patients with MSI-H, MSI-L and MSS status in COAD **(B)** and READ **(C)**, respectively. **(D)** CAFDL scores of patients with dMMR and pMMR status in GSE39582. **(E, F)** CAFDL scores of patients with MSI-H and MSS status in GSE92921 **(E)** and GSE143985 **(F)**, respectively. ns, non-significant.
