# Supplementary figures and images for: Development and validation of a cancer-associated fibroblast-derived lncRNA signature for predicting clinical outcomes in colorectal cancer
Source: Front Immunol. 2022 Jul 29;13:934221. doi: 10.3389/fimmu.2022.934221 (PMC9374325; doi:10.3389/fimmu.2022.934221)

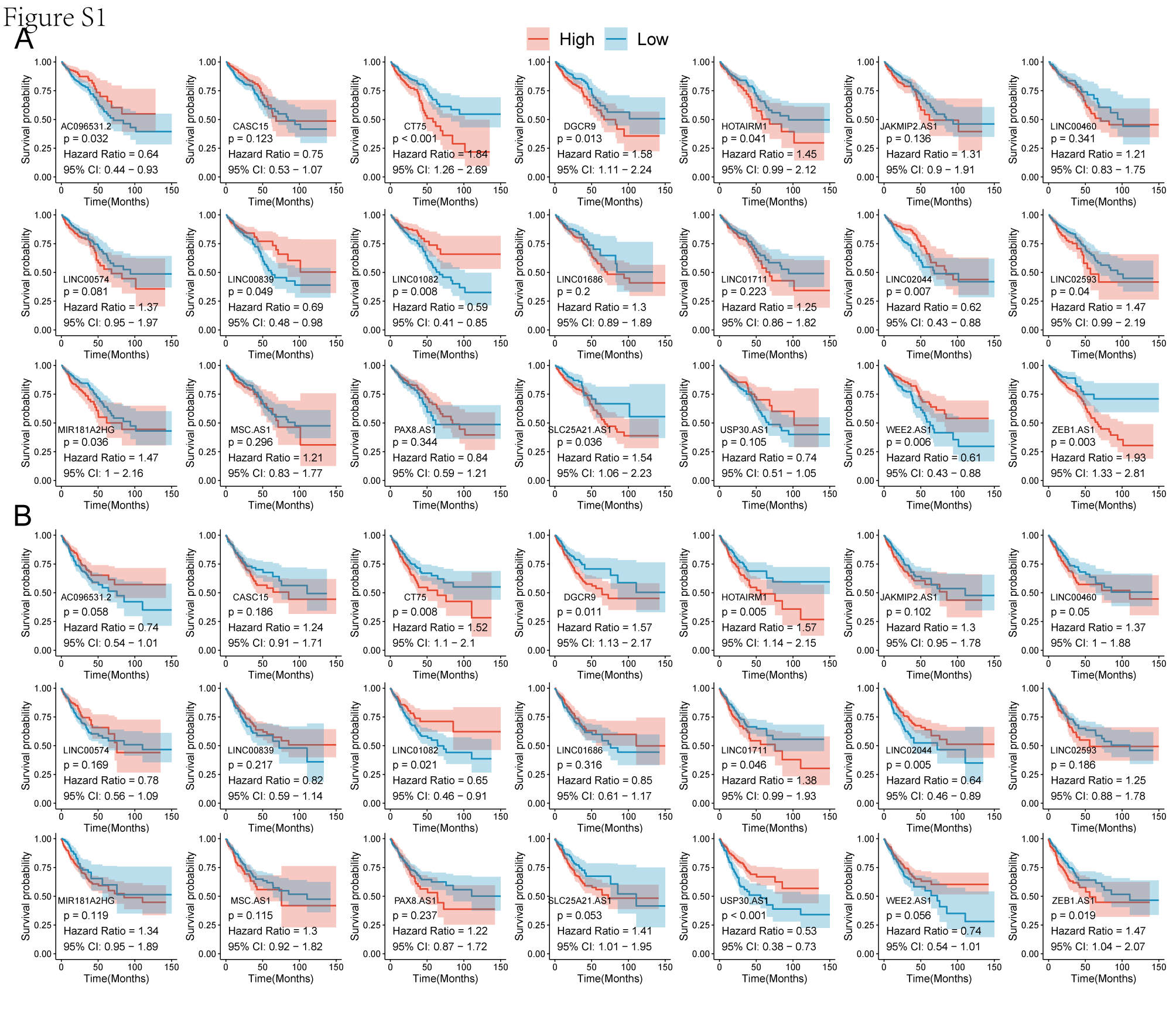

Supplement: Supplementary Figure 1 — Survival analysis for the 21 lncRNAs constituting the CAFDL signature. (A) Overall survival. (B) Disease-free survival. [file Image_1.tif]

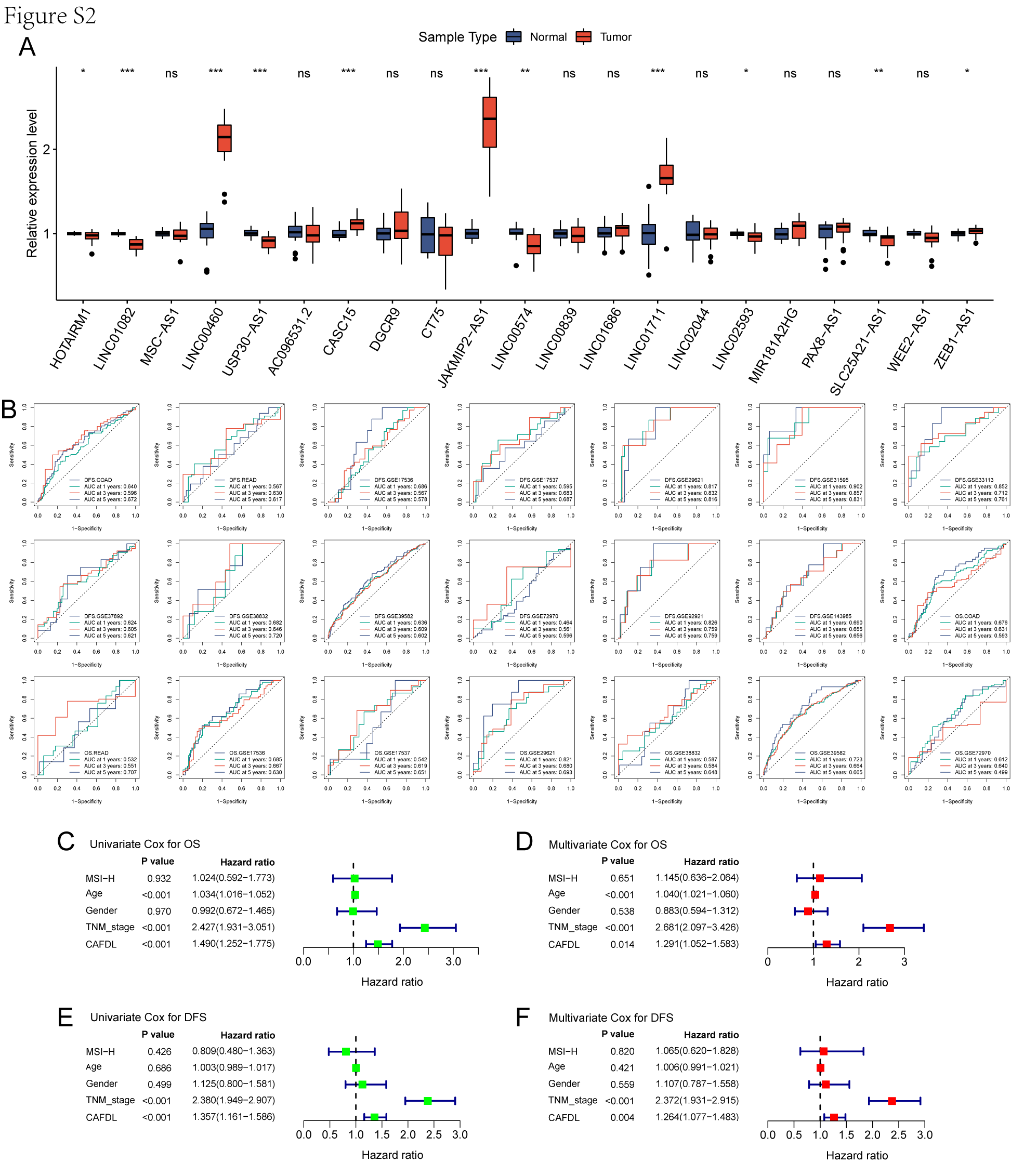

Supplement: Supplementary Figure 2 — Validation of the CAFDL signature. (A) qRT-PCR detected the expression levels of 21 lncRNAs constituting the CAFDL signature in paired CRC and adjacent normal tissues. (B) ROC analysis of the predictive ability of CAFDL signature on DFS and OS at 1, 3, and 5 years in CRC datasets. (C, D) Univariate and multivariate Cox analysis identify independent predictive factors for OS in TCGA-CRC cohort. (E, F) Univariate and multivariate Cox analysis identify independent predictive factors for DFS in TCGA-CRC cohort. [file Image_2.tif]

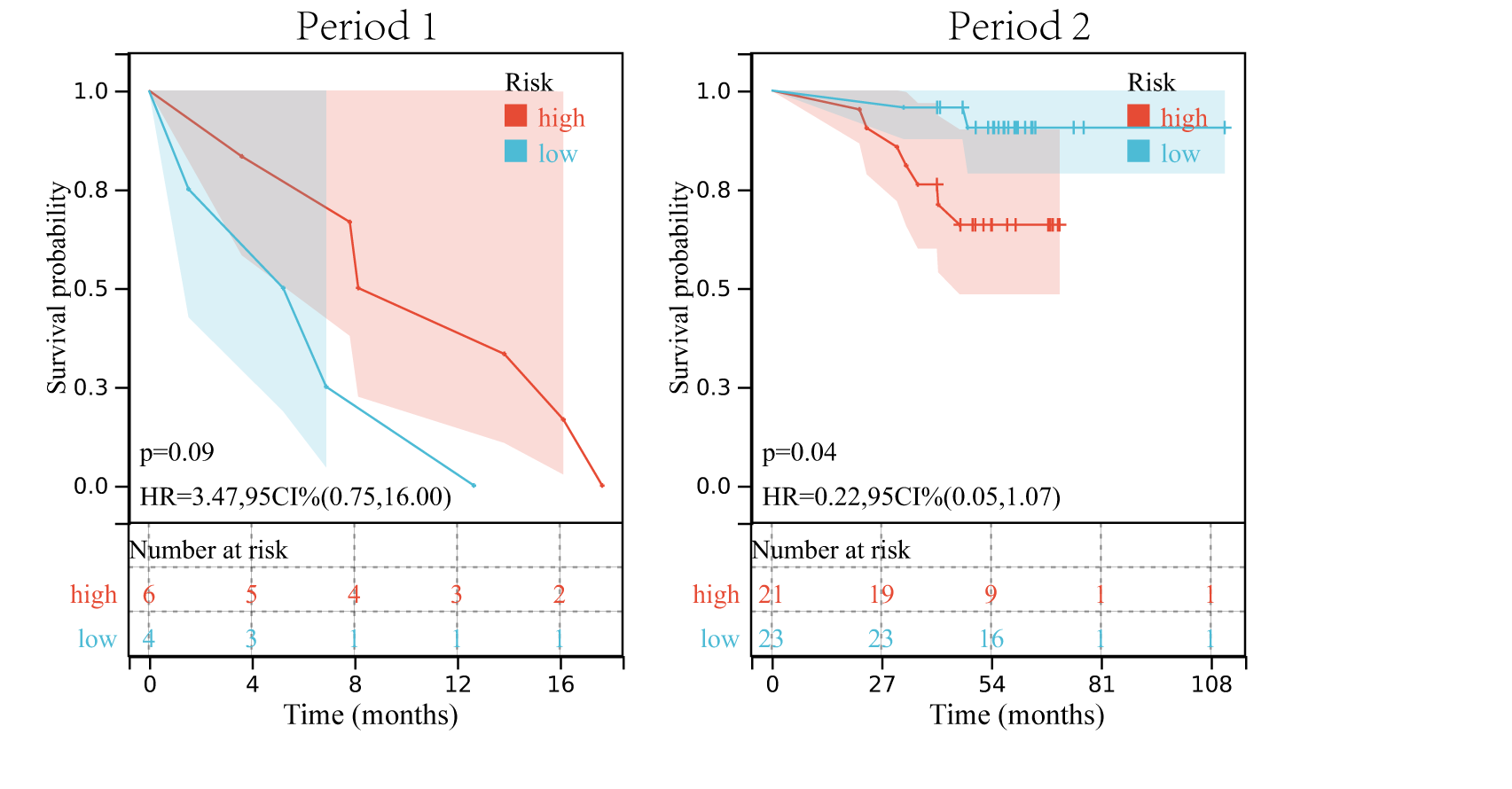

Supplement: Supplementary Figure 3 — Landmark analysis of GSE17537. [file Image_3.tif]

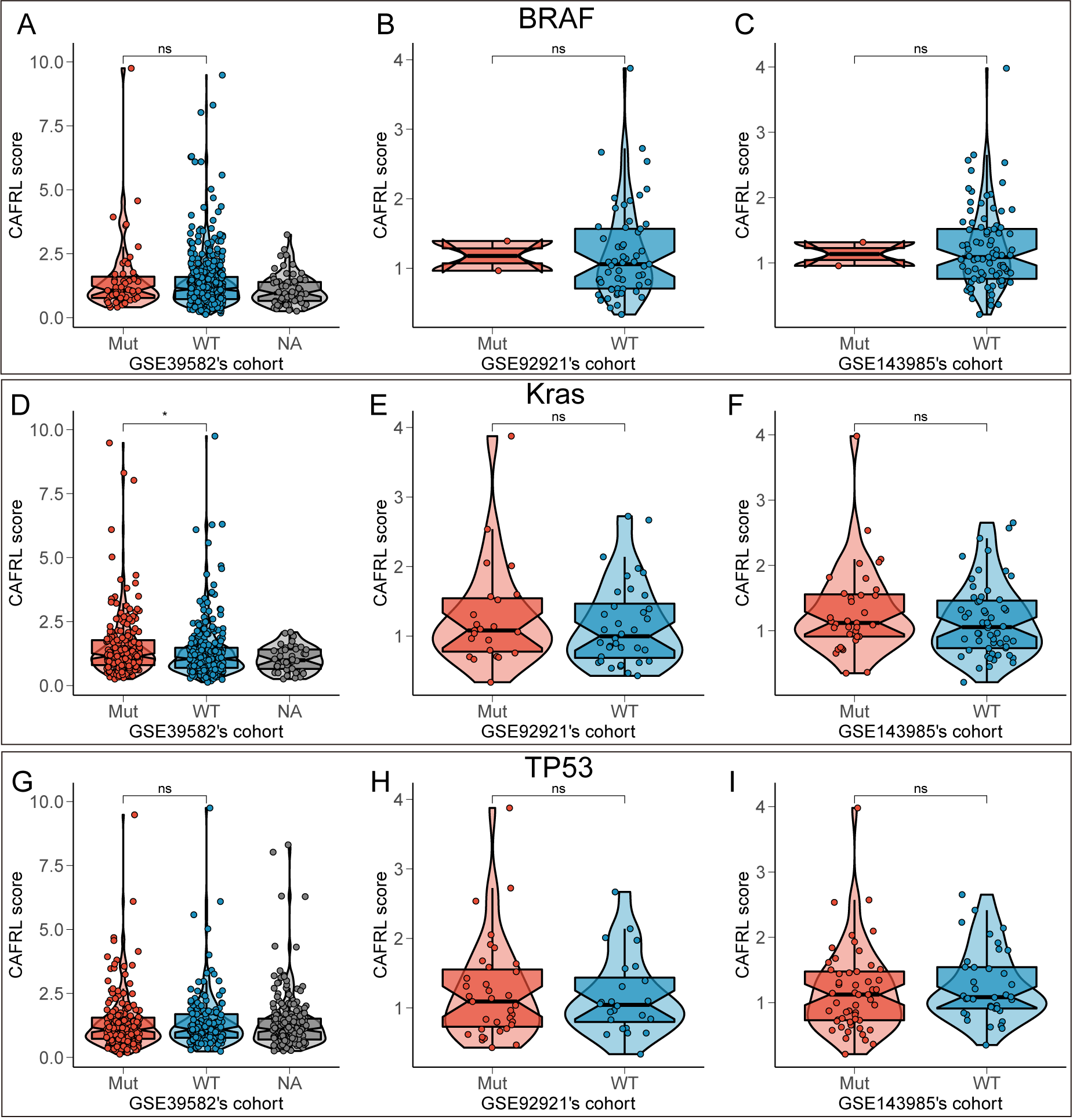

Supplement: Supplementary Figure 4 — CAFDL score in patients with wild-type or mutant BRAF, Kras and TP53. (A-C) CAFDL score in patients with wild-type or mutant BRAF in GSE39582, GSE92921 and GSE143985 cohorts. (D-F) CAFDL score in patients with wild-type or mutant Kras in GSE39582, GSE92921 and GSE143985 cohorts. (G-I) CAFDL score in patients with wild-type or mutant TP53 in GSE39582, GSE92921 and GSE143985 cohorts. ns, non-significant. [file Image_4.tif]

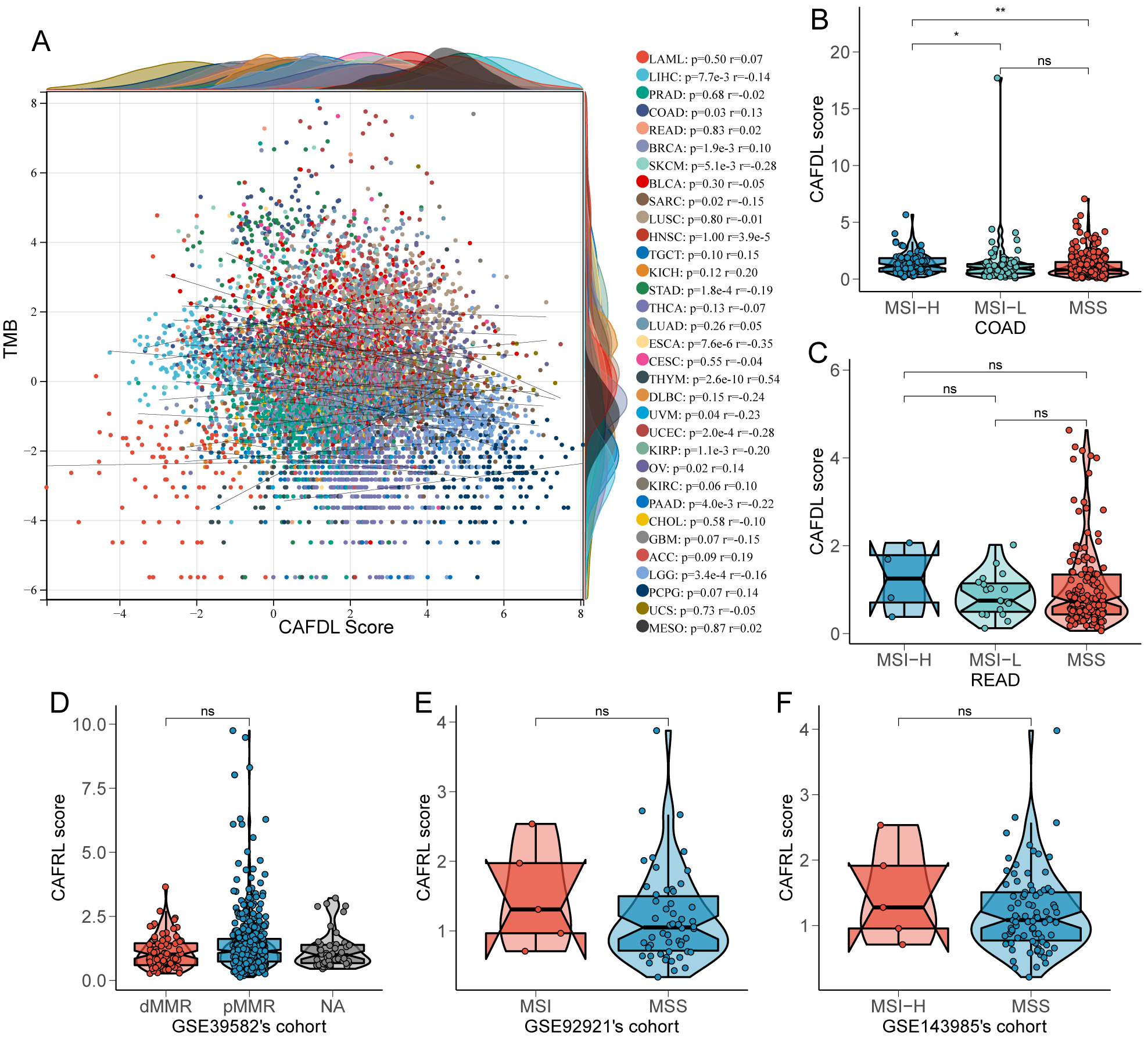

Supplement: Supplementary Figure 5 — Correlation between CAFDL signature and TMB and MSI/MMR status. (A) Correlation of CAFDL signature with TMB across pan-cancer. (B, C) CAFDL scores of patients with MSI-H, MSI-L and MSS status in COAD (B) and READ (C), respectively. (D) CAFDL scores of patients with dMMR and pMMR status in GSE39582. (E, F) CAFDL scores of patients with MSI-H and MSS status in GSE92921 (E) and GSE143985 (F), respectively. ns, non-significant. [file Image_5.tif]
